# Entomological inferences highlight the risk of *Leishmania* transmission in the urban area of Porto Velho, Rondônia, Brazil

**DOI:** 10.1371/journal.pone.0309168

**Published:** 2024-08-16

**Authors:** Michelli Santos da Silva, Amanda Maria Picelli, Kamila Pereira de França, Eunice Aparecida Bianchi Galati, José Dilermando Andrade Filho, Genimar Rebouças Julião, Felipe Dutra-Rêgo, Jansen Fernandes de Medeiros

**Affiliations:** 1 Laboratory of Entomology, Oswaldo Cruz Foundation, Fiocruz Rondônia, Porto Velho, RO, Brazil; 2 Postgraduate Program in Experimental Biology—PGBIOEXP, Fiocruz Rondônia/ UNIR, Porto Velho, RO, Brazil; 3 Department of Biology and Center for Biodiversity and Ecosystem Stewardship Villanova University, Villanova, PA, United States of America; 4 Department of Epidemiology, School of Public Health, University of São Paulo–USP, São Paulo, Brazil; 5 Leishmaniasis Study Group, Instituto René Rachou, Oswaldo Cruz Foundation (FIOCRUZ), Belo Horizonte, MG, Brazil; 6 National Institute of Epidemiology in the Western Amazon—INCT-EpI-AmO, Porto Velho, RO, Brazil; Instituto Leonidas e Maria Deane / Fundacao Oswaldo Cruz, BRAZIL

## Abstract

Entomological investigations were conducted for the first time in urban forest remnants of Porto Velho, state of Rondônia, Brazil, to explore the transmission dynamics of *Leishmania*. Sand fly collections were carried out at ten sites, encompassing both canopy and ground strata, from October to December 2021. A total of 1,671 sand flies were collected, representing 42 species within 12 genera. *Nyssomyia* Antunesi (n = 384) and *Psychodopygus davisi* (n = 111) were the most abundant species. Molecular analyses targeting the V7V8 region (18S gene) unveiled the presence of sequences 100% identical to *Leishmania infantum* in females of *Bichromomyia flaviscutellata* (1), *Nyssomyia* Antunesi complex (6), *Nyssomyia umbratilis* (1), *Nyssomyia* sp. (1), *Psychodopygus ayrozai* (1), *Ps*. *davisi* (3), *Psychodopygus paraensis* (1), and *Sciopemyia sordellii* (1). Sequences 100% similar to *Trypanosoma minasense* were found in two samples of the *Nyssomyia* Antunesi complex, and two samples of *Sc*. *sordellii* presented 100% identity to a *Trypanosoma* sp. strain, previously identified in this same sand fly in Rondônia. Sequencing of *Cytb* fragment suggested *Homo sapiens*, *Dasypus novemcinctus* and *Tamandua tetradactyla* as the blood source for distinct sand flies. The identification of sequences similar to *L*. *infantum* in sand flies collected in urban forest fragments is noteworthy, correlating with the recent local and regional occurrence of autochthonous cases of human visceral leishmaniasis. However, further studies are imperative to ascertain the presence of hosts/reservoirs and evaluate the risk of *L*. *infantum* transmission to humans.

## Introduction

Sand flies (Diptera, Psychodidae) play a pivotal role as the primary vectors of *Leishmania*, contributing to the global public health concern caused by leishmaniasis [1]. Among the countries significantly affected by both cutaneous (CL) and visceral (VL) forms of leishmaniasis, Brazil stands out, reporting high incidence rates [2]. The state of Rondônia (RO), situated in the northern region, has reported approximately 16,214 CL cases from 2007 to 2022, with sporadic occurrences of VL, totaling eight autochthonous human cases in the same period [3]. Notably, the municipalities of Porto Velho and Vilhena exhibit elevated CL-prevalence, with the disease primarily associated with rural and forested areas throughout the state [3]. Nevertheless, the epidemiological profile of CL, primary associated with sylvatic areas, has undergone changes, leading to the emergence of autochthonous cases in both rural and periurban areas [4–6].

Currently, Rondônia boasts a documented diversity of approximately 140 sand fly species [7]. After Amazonas, thus, Rondônia has the highest levels of sand fly diversity of all Brazil´s federal states [8]. Despite extensive research in rural and sylvatic areas [9, 10], the sand fly diversity in urban forest fragments remains unexplored. Consequently, our objective was to: provide the first description of the sand fly fauna in the urban area of Porto Velho; determine the food source in engorged females, and screen for the presence of Trypanosomatidae DNA.

## Material and methods

### Ethical statements

For sand fly collection, the guidelines of Normative Instruction No. 141, dated December 19, 2006, were followed, which exempts the need for authorization for collection and transportation through the Biodiversity Authorization and Information System (SISBio). Additionally, the project is registered in the National System for Management of Genetic Heritage and Associated Traditional Knowledge (SisGen) with Registration Number AA32B8E.

### Study area, collection, and identification of sand flies

**Fig 1:**
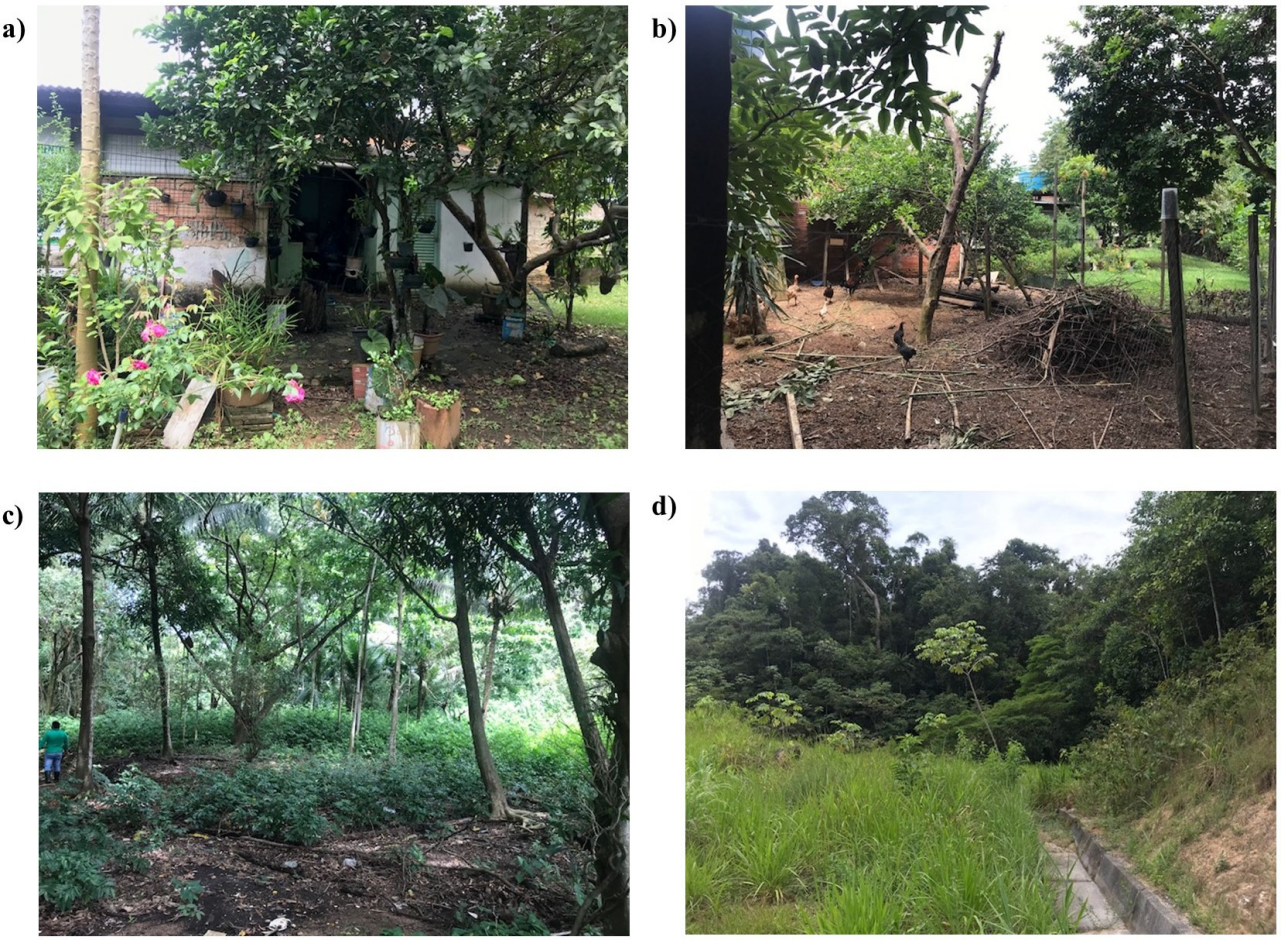
Illustrative images of some localities where traps were installed, depicting small forest fragments in urban environments and the presence of animals such as chickens. Images under a CC BY license, with permission from Michelli Santos da Silva, copyright 2023.

Sand fly collections were conducted in October, November, and December 2021 at 10 vegetation sites within the urban area of Porto Velho. Brief descriptions of the sampling locations are provided in (Table 1). Notably, two of the sites, designated as "Parque Natural" and "UNIR," are classified as urban expansion areas according to the Secretaria de Planejamento, Orçamento e Gestão (SEMPOG) of the municipality. The selection of sampling sites was based on the following criteria: (a) the presence of riparian vegetation (fragments of vegetation along riverbanks), (b) the presence of low-order streams, locally called "*igarapés*", and (c) the presence of human dwellings and/or circulation and domestic animals (Fig 1).

**Table 1:** Geographical coordinates and brief descriptions of the sampling sites in Porto Velho, RO, Brazil.

Sampling sites	Geographical coordinates	Description
Residencial Viena	8°48’38.20"S 63°50’22.50"W	2.0km^2^ size forest fragment nearby a condominium, with domestic and wild animals. Recent human habitation area.
Parque Natural R. P. de Oliveira	8°40’56.40"S 63°51’48.10"W	10.7km^2^ size forest fragment in a municipal reserve, with recreational and touristic trails (ecotourism), presence of domestic and wild animals.
UNIR	8°49’59.80"S 63°56’36.00"W	1.71km^2^ size forest fragment at the Federal University of Rondônia, with student and visitor trails, constant presence of humans, domestic and wild animals.
Vila Tupi	8°46’44.05"S 63°54’1.20"W	0.19 km^2^ size forest fragment in a neighborhood, presence of domestic and wild animals.
Ulisses Guimarães	8°46’1.00"S 63°48’15.63"W	0.75km^2^ size forest fragment in a neighborhood, with *cupuaçu* tree cultures (typical fruit from northern Brazil), domestic and wild animals.
Areia Branca	8°48’14.70"S 63°54’22.30"W	1.60km^2^ size forest fragment in a neighborhood, domestic and wild animals.
São João Batista	8°46’56.41"S 63°53’17.61"W	0.12km^2^ size forest fragment in a neighborhood, domestic and wild animals.
Nova Esperança	8°43’30.72"S 63°51’41.62"W	0.36km^2^ size forest fragment in a neighborhood, new area, and human habitation.
Agenor de Carvalho	8°45’19.99"S 63°52’13.60"W	0.04km^2^ size fragment of forest in a neighborhood, scout headquarters with protected area for a water spring and used for recreational purposes, presence of domestic and wild animals.
São João Bosco	8°44’35.54"S 63°54’5.73"W	0.1km^2^ size fragment of forest in a neighborhood, wild and domestic animals, area with solid waste disposal.

Sand flies were simultaneously collected at the ten points using HP light traps [11] adapted with green LED [10], installed for 12 hours at two different heights, covering distinct strata: one meter above the ground and in the forest canopy at an approximate height of 10 to 12 meters. After each collection, the sand flies were preserved in 92% ethanol and transported to the Laboratory of Entomology at Fiocruz Rondônia. Male sand flies were directly mounted in Berlese liquid. Female sand flies were initially screened for the presence of residual blood in the abdomen and then dissected to remove the head and the last three tergites for subsequent taxonomic identification [12]. The remaining body parts were individually stored in 92% ethanol at -20°C for molecular procedures. The abbreviations of sand fly genera and subgenera followed [13].

### Molecular detection of Trypanosomatidae

Prior to DNA extraction, non-engorged female sand flies were organized into groups or individually, each comprising up to twenty specimens from the same month, location, stratum, and species. Whole DNA extraction was performed using the Gentra Puregene Cell and Tissue Kit (Qiagen, Valencia, CA), following the manufacturer’s guidelines. To mitigate the risk of cross-contamination, all instruments and workspaces underwent decontamination with DNAZap (Ambion Life Technologies, Inc.). Additionally, male sand flies were included as negative control groups throughout all extraction steps. DNA from engorged females was individually processed using the illustra blood genomicPrep Mini Spin Kit (GE Healthcare, Piscataway, NJ), following [14] to prevent cross-contaminations.

The extracted DNA underwent purity assessment using spectrophotometry (NanoDrop), and PCR targeting the cacophony gene in the ISV6 region [15] was utilized to evaluate DNA quality and as an endogenous control for the PCR reactions.

Nested polymerase chain reaction (nested-PCR) was performed targeting the trypanosome barcode, the V7V8 region of the 18S gene [16]. In the first stage, a 927 bp fragment was amplified using the primers TRY927F (5’-GAAACAAGAAACACGGGAG-3’) and TRY927R (5’-CTACTGGGCAGCTTGGA-3’). In the second stage, a 561-bp internal fragment of V7V8 was amplified using the primers SSU561F (5’-TGGGATAACAAAGGAGCA-3’) and SSU561R (5’-CTGAGACTGTAACCTCAAAGC-3’) [17]. For all PCR assays, a reference strain of *Leishmania braziliensis* (MHOM/BR/1975/M2903) served as a positive control, and a non-template sample served as a negative control. PCR-positive products were purified using the QIAquick PCR Purification Kit (Qiagen, Valencia, CA) and subsequently subjected to Sanger sequencing [18]. The analysis of electropherograms from both forward and reverse primers was primarily conducted using Finch TV software (Geospiza, Inc., Seattle, USA). Consensus sequences were obtained through BioEdit alignment software and subsequently compared with sequences deposited in the GenBank database using the megaBLAST tool. A cut-off threshold of 98% identity and 100% sequence coverage were applied to confirm the identity of Trypanosomatidae using the V7V8 set of primers. The V7V8 sequences obtained in this study have been deposited in the GenBank database under accession numbers (OR814185-OR814205).

### Blood source identification

Engorged female sand flies, characterized by the presence of blood in the midgut, were dissected using sterile needles in PBS 1x. The head and the last three tergites were mounted in Berlese medium for subsequent taxonomic identification [12], and the remaining parts of the body were stored dry at -20°C until DNA extraction using the illustra blood genomicPrep Mini Spin Kit (GE Healthcare, Piscataway, NJ). Blood meals were identified using the set of primers *cytb1* and *cytb2*, which target a region of approximately 361 bp of the cytochrome B (*Cytb*) gene [19], the first-choice target for mammal identification [20]. DNA from *Gallus gallus* was used as a positive control, and males of laboratory-reared *Lutzomyia longipalpis* were used as a negative control to monitor cross-contaminations. PCR-positive products were purified using the QIAquick PCR Purification Kit (Qiagen, Valencia, CA) and subsequently subjected to Sanger sequencing [17]. The electropherogram analysis, construction of consensus sequences, and megaBLAST followed the same protocol described for the molecular detection of Trypanosomatidae. Additionally, a cut-off of 98% identity and 100% sequence coverage were used to ascertain the vertebrate food source of engorged females. The cytb sequences obtained in this study have been deposited in the GenBank database under accession numbers (PP957632-PP957657).

## Results

### Sand fly fauna

A total of 1,671 specimens were collected, comprising 1,055 specimens in the canopy (63.1%) and 616 on the ground (36.9%). Some females were identified only at the genus level, such as *Trichophoromyia* spp. and *Trichopygomyia* spp., while others were identified at the series level, such as *Psychodopygus* (Chagasi and Guyanensis series), due to morphological damage. Indeed, a total of 1,631 specimens (97.6%) were identified at least at the genus level, comprising 42 species belonging to 12 genera (Table 2).

**Table 2:** Sand flies species collected in the canopy and ground strata in the urban forest remnants of Porto Velho, Rondônia, in 2021.

Species	Strata				
	Canopy		Ground		Total (%)
	Female	Male	Female	Male	
*Bichromomyia flaviscutellata*	3	2	11	1	17 (1.04)
*Evandromyia brachyphalla*	-	-	1	-	1 (0.06)
*Evandromyia georgii*	-	-	6	1	7 (0.43)
*Evandromyia infraspinosa*	1	-	-	-	1 (0.06)
*Evandromyia piperiformis*	-	-	4	-	4 (0.25)
*Evandromyia saulensis*	2	1	7	2	12 (0.74)
*Evandromyia* (Infraspinosa series) sp.*	-	-	1	-	1 (0.06)
*Evandromyia* sp.*	2	-	3	-	5 (0.31)
*Evandromyia tarapacaensis*	-	1	-	2	3 (0.18)
*Evandromyia walkeri*	2	2	3	1	8 (0.49)
*Evandromyia wilsoni*	-	1	4	-	5 (0.31)
*Lutzomyia* (*Tricholateralis*) sp.	-	-	3	-	3 (0.18)
*Lutzomyia evangelistai*	-	1	-	-	1 (0.06)
*Lutzomyia sherlocki*	6	23	5	2	36 (2.21)
*Lutzomyia* sp.*	1	-	6	-	7 (0.43)
*Micropygomyia* sp. (Pilosa series) *	1	-	-	-	1 (0.06)
*Nyssomyia antunesi*	-	323	-	61	384 (23.54)
*Nyssomyia* Antunesi complex	266	-	68	-	334 (20.48)
*Nyssomyia fraihai*	7	14	1	3	25 (1.53)
*Nyssomyia richardwardi*	2	-	-	-	2 (0.12)
*Nyssomyia shawi*	1	-	-	-	1 (0.06)
*Nyssomyia* sp.*	9	1	2	-	12 (0.74)
*Nyssomyia umbratilis*	4	5	2	1	12 (0.74)
*Nyssomyia urbinattii*	-	-	-	1	1 (0.06)
*Pintomyia fiocruzi*	-	1	-	1	2 (0.12)
*Pintomyia odax*	-	1	-	-	1 (0.06)
*Pintomyia* sp. (Serrana series) *	1	2	2	-	5 (0.31)
*Pintomyia* sp.*	-	-	-	1	1 (0.06)
*Psathyromyia aragaoi*	1	1	1	2	5 (0.31)
*Psathyromyia barrettoi*	-	-	-	1	1 (0.06)
*Psathyromyia dreisbachi*	1	-	-	-	1 (0.06)
*Psathyromyia lutziana*	-	-	1	1	2 (0.12)
*Psathyromyia* (Shannoni series)	-	-	1	-	1 (0.06)
*Psathyromyia* (*Xiphopsathyromyia*) sp.*	-	-	1	-	1 (0.06)
*Psathyromyia* sp.*	1	-	1	-	2 (0.12)
*Psychodopygus amazonensis*	-	3	1	-	4 (0.25)
*Psychodopygus ayrozai*	10	21	21	11	63 (3.86)
*Psychodopygus carrerai*	7	8	1	4	20 (1.23)
*Psychodopygus chagasi*	-	26	-	3	29 (1.78)
*Psychodopygus claustrei*	9	23	3	12	47 (2.9)
*Psychodopygus complexus*	-	16	-	2	18 (1.10)
*Psychodopygus davisi*	34	52	15	10	111 (6.81)
*Psychodopygus paraensis*	6	5	9	-	20 (1.23)
*Psychodopygus* sp. (Chagasi series) *	57	-	27	-	84 (5.15)
*Psychodopygus* sp. (Guyanensis series) *	18	-	4	-	22 (1.35)
*Psychodopygus* sp.*	3	-	1	-	5 (0.31)
*Sciopemyia sordellii*	1	5	35	19	58 (3.56)
*Sciopemyia* sp.*	4	1	14	-	21 (1.29)
*Thrichophoromyia octavioi*	-	-	-	1	1 (0.06)
*Trichophoromyia auraensis*	-	4	-	21	25 (1.53)
*Trichophoromyia clitella*	-	1	-	34	35 (2.15)
*Trichophoromyia howardi*	-	-	-	3	3 (0.18)
*Trichophoromyia* sp.*	2	-	22	1	25 (1.53)
*Trichophoromyia ubiquitalis*	4	4	9	26	43 (2.64)
*Trichopygomyia dasypodogeton*	-	6	-	19	25 (1.53)
*Trichopygomyia rondoniensis*	-	1	-	12	13 (0.80)
*Trichopygomyia* sp.	9	-	36	-	45 (2.76)
*Trichopygomyia trichopyga*	-	-	-	4	4 (0.25)
*Viannamyia furcata*	1	1	-	-	2 (0.12)
*Viannamyia* sp.*	1	-	1	-	2 (0.12)
*Viannamyia tuberculata*	-	-	2	-	2 (0.12)
**Total (%)**	**477**	**556**	**335**	**263**	**1,631**
	**1,033 (63)**		**598 (37)**		

* Specimens with morphological characters damaged.

*Evandromyia* and *Psychodopygus* were the genera more frequently captured with eight species each, followed by *Nyssomyia* (six species) and *Psathyromyia* (five species). However, eight more genera have been recorded in forest fragments within the urban areas: *Bichromomyia* (one species), *Lutzomyia* (two species), *Micropygomyia* (at least one species of Pilosa series), *Pintomyia* (two species), *Sciopemyia* (one species), *Trichophoromyia* (five species), *Trichopygomyia* (three species), and *Viannamyia* (two species) (Table 2). The most frequent and abundant species were *Nyssomyia antunesi* (n = 384) and *Psychodopygus davisi* (n = 111).

Among the collection locations, the highest abundance of specimens was found in Parque Natural (n = 555, 33%), followed by Areia Branca (n = 389, 23%), UNIR (n = 226, 14%), Residencial Viena (n = 200, 12%), Nova Esperança (n = 126, 8%), Vila Tupi (n = 88, 5%), Agenor de Carvalho (n = 73, 4%), Ulisses Guimarães (n = 8, 0%), and São João Bosco (n = 2, 0%). No sand flies collected in São João Batista.

### Molecular detection of Trypanosomatidae

A total of 759 non-engorged female sand flies were screened for the presence of Trypanosomatidae DNA. Among these, 152 specimens were individually analyzed, while the remaining were grouped into 142 pools, resulting in a total of 294 samples. The amplification of the 561 bp of V7V8 fragment yielded positive results in 19 samples, accounting for 7.14% of the total. Fifteen out of the 19 positive samples exhibited similarity to *L*. *infantum* and were obtained from the following species: *Br*. *flaviscutellata* (1), *Nyssomyia* sp. (1), *Ny*. Antunesi complex (6), *Ny*. *umbratilis* (1), *Ps*. *ayrozai* (1), *Ps*. *davisi* (3), *Ps*. *paraensis* (1), and *Sc*. *sordellii* (1). All *L*. *infantum* sequences were 100% identical to *L*. *infantum* sequences previously identified in Cuiabá, state of Mato Grosso, Brazil (Accession number MH410817.1).

Four samples exhibited similarity to the *Trypanosoma* genus. Among these, two samples of the *Ny*. Antunesi complex exhibited 100% similarity to *Trypanosoma minasense*, previously identified in Madre de Dios region, Peru (Accession number KX932489.1) [21]. The remaining two samples, belonging to *Sc*. *sordellii*, had 100% similarity to *Trypanosoma* sp. This trypanosome has previously been identified in the same city (Porto Velho) within the same sand fly [16] (Accession number EU021243.1). All trypanosomatid findings are summarized in Table 3.

**Table 3:** Molecular detection of Trypanosomatidae in females collected in October, November, and December 2021 in forest fragments in the urban area of Porto Velho, Rondônia, Brazil.

Species	Total of individual / Pool samples		Positivity for *Leishmania/Trypanosoma*	
	Canopy	Ground level	Canopy	Ground level
*Bi*. *flaviscutellata*	3/0	4/2	-	1/0
*Ev*. *brachyphalla*	0/1	0/0	-	-
*Ev*. *georgii*	0/4	0/0	-	-
*Ev*. *infraspinosa*	1/0	0/0	-	-
*Ev*. *piperifomis*	0/1	0/0	-	-
*Ev*. *saulensis*	2/0	3/1	-	-
*Evandromyia*. (Infraspinosa series)	0/0	1/0	-	-
*Evandromyia sp*.	0/1	0/1	-	-
*Ev*. *walkeri*	1/0	0/1	-	-
*Ev*. *wilsoni*	0/0	3/0	-	-
*Lu*. *sherlocki*	3/1	3/1	-	-
*Lu*. (*Tricholateralis)* sp.	0/0	1/0	-	-
*Lutzomyia* sp.	1/0	3/1	-	-
*Micropygomyia*. sp. (Pilosa series)	1/0	0/0	-	-
*Ny*. Antunesi complex	7/34	10/10	5/0	1/2*
*Ny*. *frahai*	4/1	1/0	-	-
*Ny*. *richardwardi*	1/0	0/0	-	-
*Ny*. *shawi*	1/0	0/0	-	-
*Nyssomyia* sp.	3/2	2/0	1/0	-
*Ny*. *umbratilis*	1/1	1/0	1/0	-
*Pintomyia* sp. (Serrana series)	1/0	2/0	-	-
*Pa*. *aragaoi*	1/0	1/0	-	-
*Pa*. *dreisbachi*	1/0	0/0	-	-
*Pa*. *lutziana*	1/0	0/0	-	-
*Psathyromyia* (*Xiphopsathyromyia*) sp.	0/1	0/0	-	-
*Psathyromyia* sp.	1/0	1/0	-	-
*Ps*. *amazonensis*	0/0	1/0	-	-
*Ps*. *ayrozai*	6/1	3/4	-	1/0
*Ps*. *carrerai*	2/1	1/0	-	-
*Ps*. *claustrei*	1/2	0/2	-	-
*Ps*. *davisi*	4/6	7/4	3/0	-
*Ps*. *paraensis*	4/1	1/2	-	1/0
*Psychodopygus* sp. (Chagasi series)	3/10	6/5	-	-
*Psychodopygus* sp. (Guyanenisis series)	6/2	4/0	-	-
*Psychodopygus* sp.	0/1	1/0	-	-
*Sc*. *sordeliii*	1/0	7/11	0/1**	1/1**
*Sciopemyia* sp.	4/2	6/1	-	-
*Trichophoromyia* sp.	2/0	5/4	-	-
*Th*. *ubiquitalis*	4/0	3/2	-	-
*Trichopygomyia* sp.	5/2	2/5	-	-
*Vi*. *furcata*	1/0	0/0	-	-
*Vi*. *tuberculata*	1/0	1/0	-	-
*Viannamyia* sp.	1/0	1/0	-	-
**Total**	78/75	74/67	10/1	5/3

* Two Samples from *Ny*. Antunesi complex positive for *T*. *minasense* DNA.

** Two samples from *Sc*. *sordellii* positive for *Trypanosoma* sp. DNA.

The positive samples from *L*. *infantum* were found in sand flies collected in the Parque Natural (*Bi*. *flaviscutellata*, *Nyssomyia* sp., *Ny*. Antunesi complex, *Ps*. *davisi*), Areia Branca (*Ny*. Antunesi complex, *Ny*. *umbratilis* and *Sc*. *sordellii*), Nova Esperança (*Ny*. Antunesi complex), Agenor de Carvalho (*Ny*. Antunesi complex), Residencial Viena (*Ps*. *davisi*) and Vila Tupi (*Ps*. *ayrozai*, *Ps*. *paraensis* and *Sc*. *sordellii*). *Trypanosoma minasense* was found in female of the *Ny*. Antunesi complex collected in Nova Esperança and Areia Branca, and *Trypanosoma* sp. was found in *Sc*. *sordellii* collected in the Vila Tupi.

### Blood meal analysis

Of the 814 females collected, 29 (3.56%) exhibited signals of recent blood meals, with 16 specimens collected in the canopy (55%) and 13 females at ground level (45%) (Table 4). The *Ny*. Antunesi complex was the most frequently found engorged specimens (N = 7; 24.1%), followed by *Ev*. *piperiformes*, *Ps*. *ayrozai* and *Psychodopygus* series Chagasi (N = 3; 10.3% each). A total of three out of 29 samples, belonging to the *Nyssomyia* Antunesi complex, *Ps*. *claustrei*, and *Ps*. *davisi*, were Cytb PCR-negative, and the blood source was not assessed. This may be explained by the low amount of DNA or even the advanced digestion process, which is described as a limiting factor in detecting blood sources in sand flies [22]. *Homo sapiens* DNA (N = 20; 77.0%) was found associated with eleven sand fly species, with all sequences presenting 100% similarity to human sequences (Accession number OR608756) identified in a previous study focused on ascertaining the blood source of sand flies [23]. Molecular identification of *Dasypus novemcinctus* (nine-banded armadillo) (N = 3; 11.5%) was observed in two samples of *Psychodopygus ayrozai* and in one sample of *Trichophoromyia* sp. One female of *Ps*. *ayrozai* presented a mixed food source (*H*. *sapiens*/*D*. *novemcinctus*). All *D*. *novemcinctus* sequences were 100% identical to those from the same vertebrate previously identified in the state of Rondônia (Accession number MZ447989) [24]. Three samples (two belonging to the *Nyssomyia* Antunesi complex and one of the Chagasi series of the *Psychodopygus* genus) tested positive for *Tamandua tetradactyla* (lesser anteater) DNA, and all sequences had 100% similarity to *Tamandua tetradactyla* sequences (Accession number MW752264) identified in a previous study focused on ascertaining the blood source of Culicoides from Rondônia [25].

**Table 4:** Engorged females collected in canopy and ground strata in forest fragments in the urban area of Porto Velho, Rondônia, Brazil.

Species	Strata		Total (%)	Food source			Positivity for *Trypanosomatidae*
	Canopy	Ground		*H*. *sapiens*	*D*. *novemcinctus*	*T*. *tetradactyla*	
*Evandromyia georgii*	-	2	2 (7)	2	-	-	-
*Evandromyia piperiformis*	-	3	3 (10.3)	3	-	-	-
*Evandromyia walkeri*	-	1	1 (3.4)	1	-	-	-
*Nyssomyia* Antunesi complex	5	3	8 (27.7)	5	-	2	*Trypanossoma minasense* ^*a*^
*Nyssomyia fraihai*	1	-	1 (3.4)	1	-	-	-
*Nyssomyia richardwardi*	1	-	1 (3.4)	1	-	-	-
*Nyssomyia umbratilis*	-	1	1 (3.4)	1	-	-	-
*Psychodopygus ayrozai*	1	2	3 (10.3)	2^b^	2^b^	-	-
*Psychodopygus carrerai*	2	-	2 (7)	1	-	-	-
*Psychodopygus claustrei*	1	-	1 (3.4)	-	-	-	-
*Psychodopygus davisi*	2	-	2 (7)	1	-	-	-
*Psychodopygus* sp. (Chagasi series)	3	-	3 (10.3)	2	-	1	-
*Trichophoromyia* sp.	-	1	1 (3.4)	-	1	-	-
**Total (%)**	**16 (55)**	**13 (45)**	**29 (100)**	**20**	**3**	**3**	

^*a*^ The two females of *Ny*. *antunesi* complex positive for *T*. *minasense* fed on *Tamandua tetradactyla* and *Homo sapiens*.

^*b*^ One female of *Ps*. *ayrozai* presented mixed food source (*H*. *sapiens*/*D*. *novemcintus*).

Two engorged females also tested positive in V7V8-PCR, and the molecular identification revealed the presence of *Trypanosoma minasense* DNA in females of the *Nyssomyia* Antunesi complex that fed on *T*. *tetradactyla* and *H*. *sapiens*.

## Discussion

This study represents the first characterization of the sand fly fauna in urban forest remnants of Porto Velho, Rondônia, providing insights into species diversity and the potential risk of *Leishmania* transmission to humans in urban and periurban locations. Although all species collected in this study have been previously documented in Rondônia [7], this investigation highlights the presence of various vector species in the urban area, some of them presenting major or minor epidemiological significance.

The urban sand fly fauna of Porto Velho comprised at least 42 species, accounting for approximately 31% of the fauna documented in Rondônia. This may indicate the adaptive potential of several species to urban vegetation areas, particularly in forest fragment sites where domestic and wild mammals are frequently observed. The most dominant species were *Nyssomyia antunesi* and *Psychodopygus davisi*, collectively representing approximately 50% of the sand fly fauna. Both species were previously observed in abundance in studies conducted in the periurban area of Porto Velho and various locations across Rondônia [9, 10, 26]. Regarding *Ny*. *antunesi*, in various regions within the Amazonian territory, this species has shown a tendency to establish in human-modified environments [27, 28]. The precise identification of females of *Ny*. *antunesi* remains a challenge. Due to the presence of *Ny*. *urbinattii*, which, together with *Ny*. *antunesi* and *Ny*. *delsionatali* constitutes the ‘*Nyssomyia* Antunesi complex’, several females collected in this study were characterized belonging to this complex.

Several species recorded in the urban area of Porto Velho possess epidemiological relevance in the transmission of *Leishmania* sp., including *Br*. *flaviscutellata*, *Ny*. *antunesi*, *Ny*. *umbratilis*, and several species within the *Psychodopygus* genus [1]. This highlights the potential risk for humans and other host/reservoir species, especially concerning VL. However, notably, fifteen samples showed similarity to *L*. *infantum* DNA. The state of Rondônia reported eight autochthonous human cases of VL between 2007 and 2022, with one documented case in Porto Velho. Therefore, the finding of sand flies carrying *L*. *infantum* DNA is of utmost importance and prompts consideration of the possibility of a VL transmission cycle in the urban area of Porto Velho.

The occurrence of a sporadic case of VL in Porto Velho, coupled with the identification of PCR-positive sand flies, raises several epidemiological questions that warrant further investigation. Is VL truly sporadic or possibly underestimated in Porto Velho? Despite Brazil’s robust disease notification system (Sistema de Informação de Agravos de Notificação), there is a recognized fragility in the data, considering the potential for underreporting. Another crucial question pertains to the potential host/reservoir. In Brazil, the domestic dog (*Canis lupus familiaris*) is considered the primary reservoir of *L*. *infantum*. Until today, no serological survey has been conducted in Porto Velho to confirm the presence of canine VL. Additionally, no other mammal, whether synanthropic or sylvatic, has been identified as a potential host of *L*. *infantum* in the area. Moreover, it is also vital to investigate the potential of the PCR-positive sand fly species in sustaining late-stage infections of *L*. *infantum* to determine their role as vectors. Therefore, this study represents a crucial step toward understanding the dynamics of *Leishmania* transmission in the urban area of Porto Velho. Further studies with diverse focuses are strongly encouraged to deepen our knowledge in this regard.

Beyond *Leishmania* parasites, this study identified other trypanosomatids carried by female sand flies. The use of the V7V8 region, a trypanosome barcode [16, 29, 30], as a molecular target allowed the identification of *Trypanosoma* sp. DNA in females of *Sc*. *sordellii*. Here, the parasite exhibited 100% similarity with *Trypanosoma* sp. deposited in GenBank under accession number EU021243.1. According to the deposit information, this trypanosome was isolated from *Sc*. *sordellii* in Rondônia [16], similar to our observations, where the sand fly species and the study area were the same. *Sciopemyia sordellii* is known to feed on ectothermic animals, particularly frogs [31], and its role as a *Leishmania* sp. vector is a subject of debate. Although there is no conclusive evidence regarding its putative capacity to sustain late-stage infections with *Leishmania*, several studies have identified *Sc*. *sordellii* carrying *Leishmania* DNA [9, 32–34]. In this study, *L*. *infantum* was detected in *Sc*. *sordellii*, consistent with previous findings in this sand fly species in the state of Maranhão [34]. In periurban areas of Porto Velho, *Sc*. *sordellii* has also been found carrying *Leishmania naiffi* DNA [9].

*Trypanosoma minasense* was found in two samples of the *Ny*. Antunesi complex. This parasite belongs to the subgenus *Megatrypanum*, is a member of the *T*. *irwini* clade, and is known to be a simian trypanosome specie*s* [35], originally described from black-penciled marmosets (*Callithrix penicillata*) in Brazil [36]. The species has been molecularly detected in several wild primates in South America, especially in saddleback tamarin (*Leontocebus weddelli*) in southeastern Peru [21], in wild howler monkeys (*Alouatta caraya*) in northeastern Argentina [37], and in a South American red-handed tamarin (*Saguinus midas*) [38]. The sequence of V7V8 The sequence of the V7V8 fragment showed that *T*. *minasense* from Rondônia was 100% identical to Peruvian isolates (Accession number KX932489.1) [21], and the geographical proximity between these areas may explain the similarity.

The detection of trypanosomes in sand flies are often by-product of epidemiological studies on leishmaniasis. Various sand fly species have been reported infected with trypanosomes; however, few parasites have been isolated and well-characterized. The lack of knowledge regarding the food habits of sand fly species hampers the association with potential hosts and consequently, trypanosome species. Sand flies belonging to the genera *Evandromyia*, *Lutzomyia*, *Psathyromyia*, and *Sciopemyia*, captured in soil and tree trunks, have been reported to be naturally infected with anuran *Trypanosoma* DNA in the state of Rondônia [16]. Circumstantial evidence has suggested that neotropical sand flies may be vectors of rodent and bat trypanosomes of the subgenus *Megatrypanum*. *Trypanosoma freitasi* has been found naturally infecting *Psychodopygus claustrei* from Amazonas, Brazil [39], and triatomine bugs appear to be refractory to this parasite [40].

Most of the engorged females belonged to the *Nyssomyia* Antunesi complex and were found in both canopy and ground strata, similarly to reported in the state of Pará, where this species appears to switch between those strata, probably to feed on different vertebrates, indicating its eclectic feeding habit [41]. Five out of seven (71%) of engorged females of *Ny*. Antunesi complex fed on *H*. *sapiens*, and the remaining fed on *T*. *tetradactyla*. Notably, the two females of *Ny*. Antunesi complex positive for *T*. *minasense* fed on *T*. *tetradactyla* and *H*. *sapiens*, both collected in the canopy. The remaining species *Ev*. *georgii*, *Ev*. *piperirformis*, *Ev*. *walkeri*, *Ny*. *fraihai*, *Ny*. *richardwardi*, *Ny*. *umbratilis*, *Ps*. *ayrozai*, *Ps*. *carrerai*, *Ps*. *davisi*, and *Psychodopygus* series Chagasi fed on *H*. *sapiens*. Several of these species have had their food habits identified for the first time, and further studies are necessary to ascertain their capacity to feed on other vertebrates. *Psychodopygus davisi* and *Ps*. *ayrozai* have been previously reported in studies in the state of Rondônia feeding on humans [42], and the latter species was detected with mixed feeding on *D*. *novemcictus* and *H*. *sapiens*, indicating the opportunistic behavior of the species.

This study provided valuable information on the diversity of sand fly species and potential risks of *Leishmania* transmission to humans and other hosts/reservoir in urban areas of Porto Velho. The report of *L*. *infantum* DNA in sand flies indicates the need to carry out a survey to identify potential hosts//reservoirs in the study area. The high abundance of *Ny*. *antunesi* and *Ps*. *davisi* suggests an adaptation of these species to human-modified environments. The presence of these species in urban areas is particularly relevant due to their potential as vectors of *Leishmania* sp., highlighting the risk to human and animal health. Furthermore, the detection of *T*. *minasense* DNA in females of the *Ny*. Antunesi complex emphasizes the complexity of parasitic interactions in this ecosystem.
